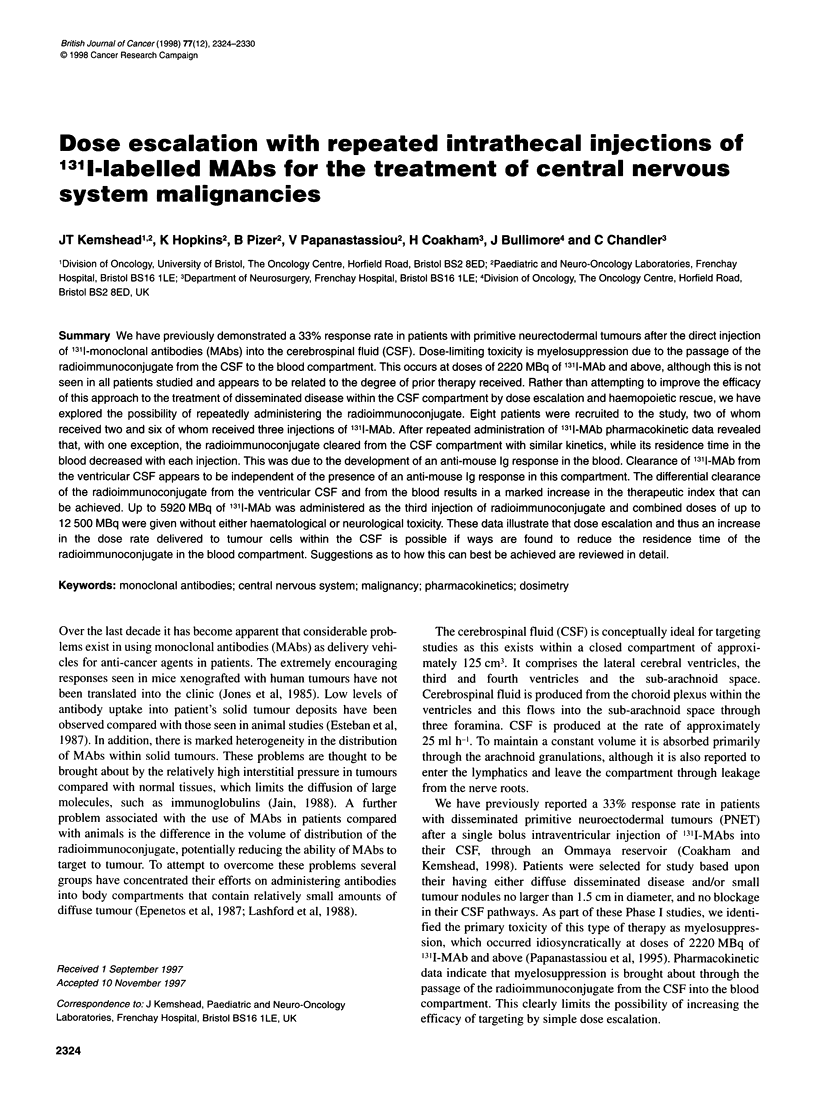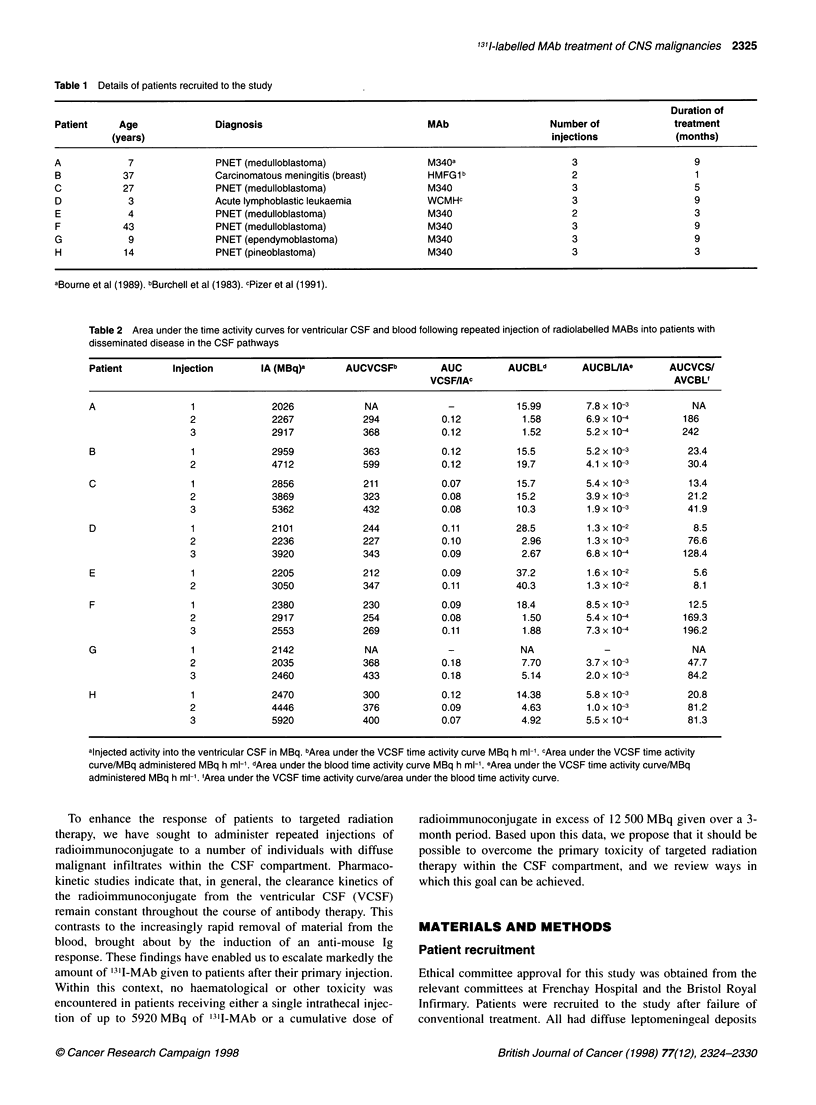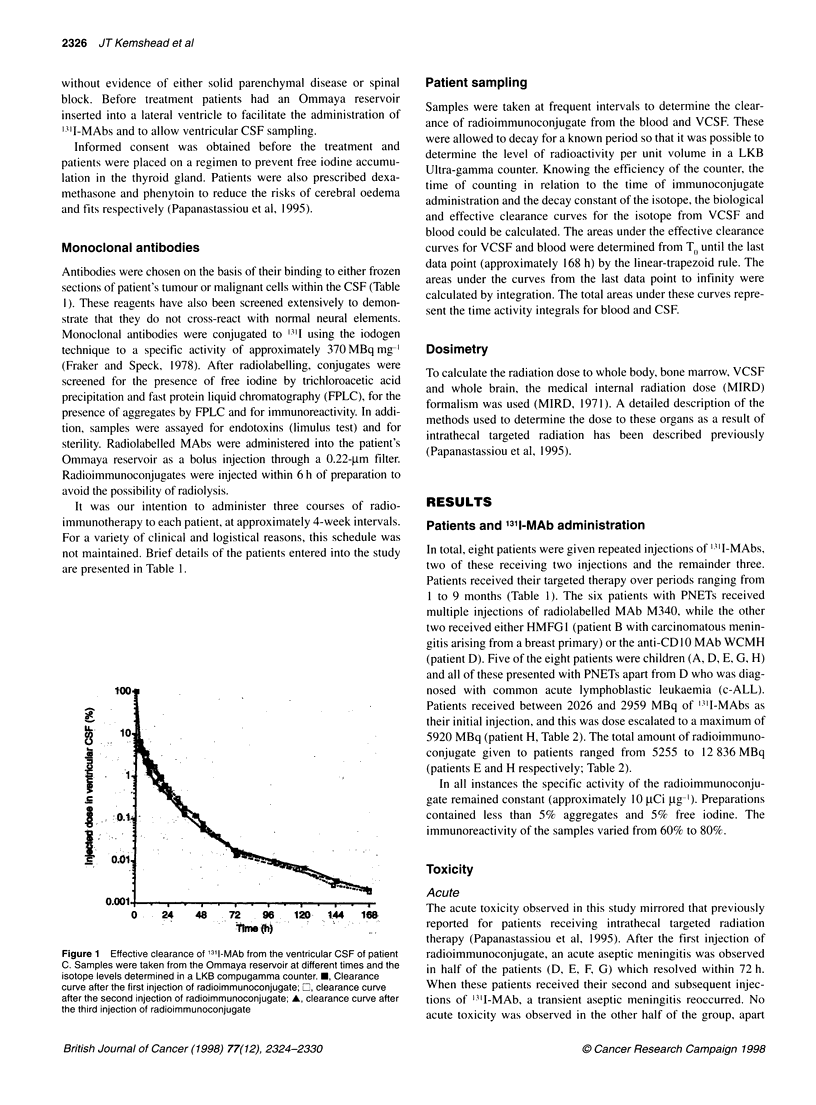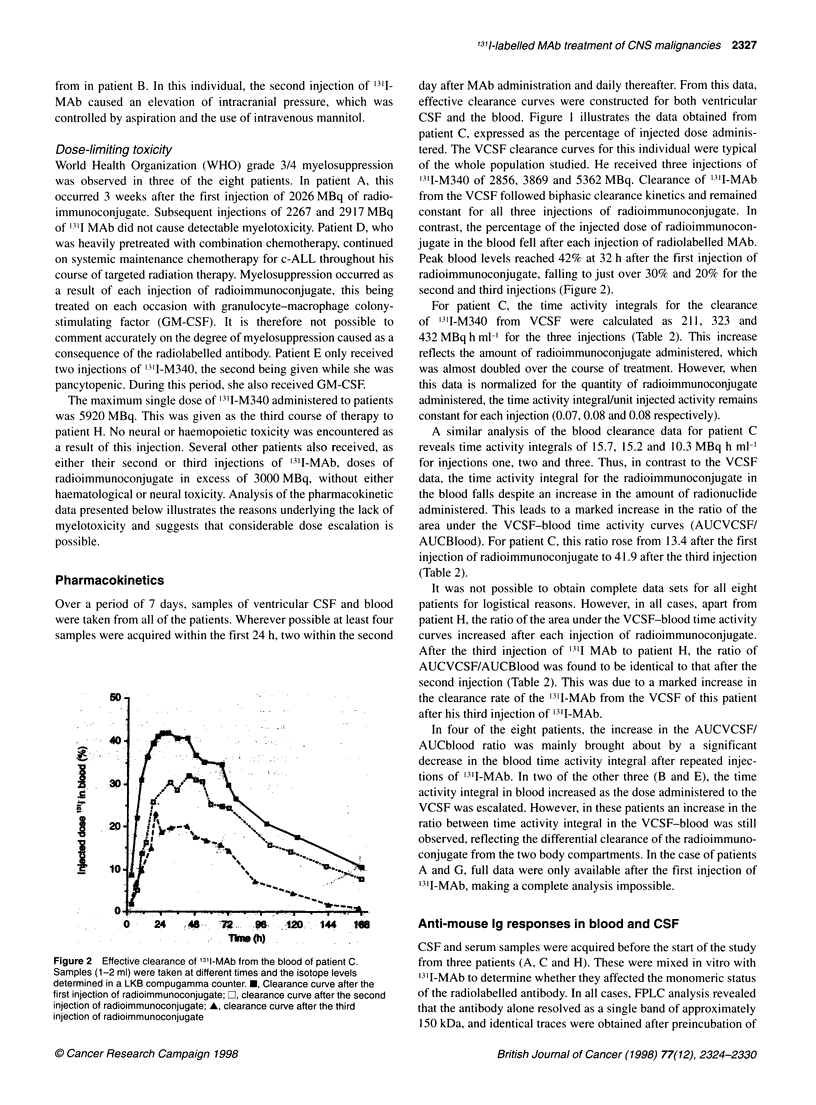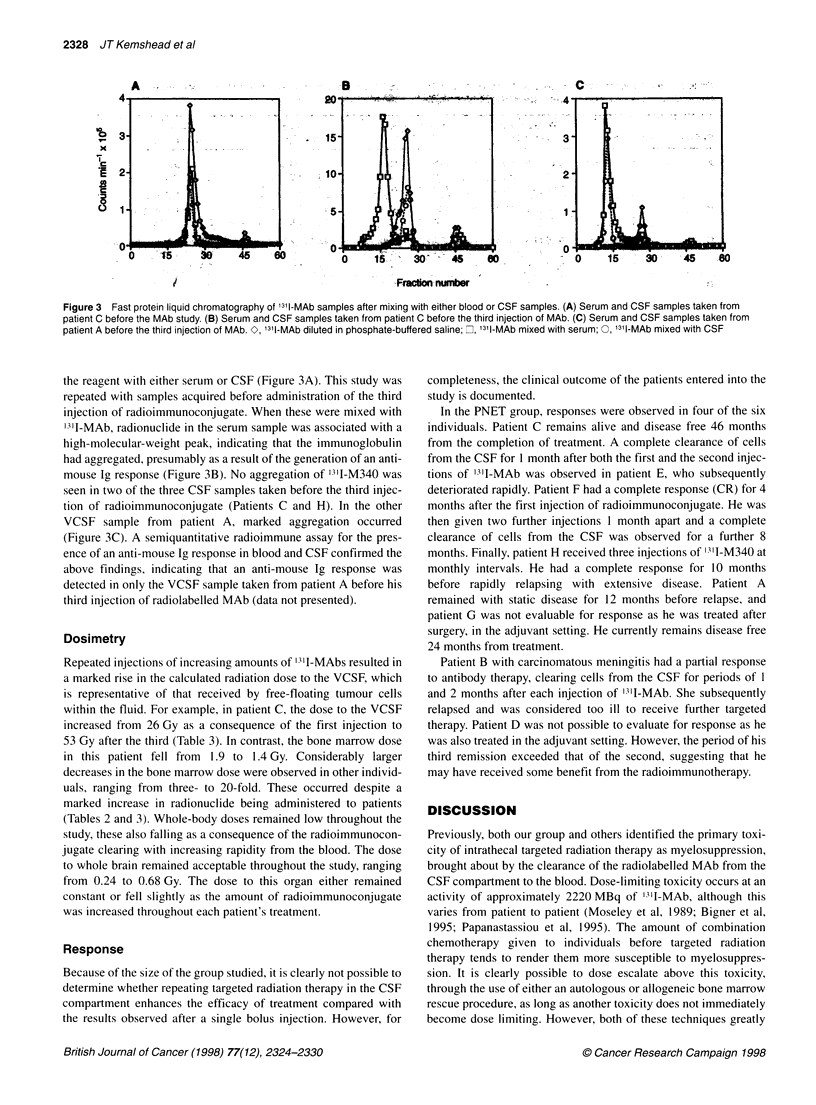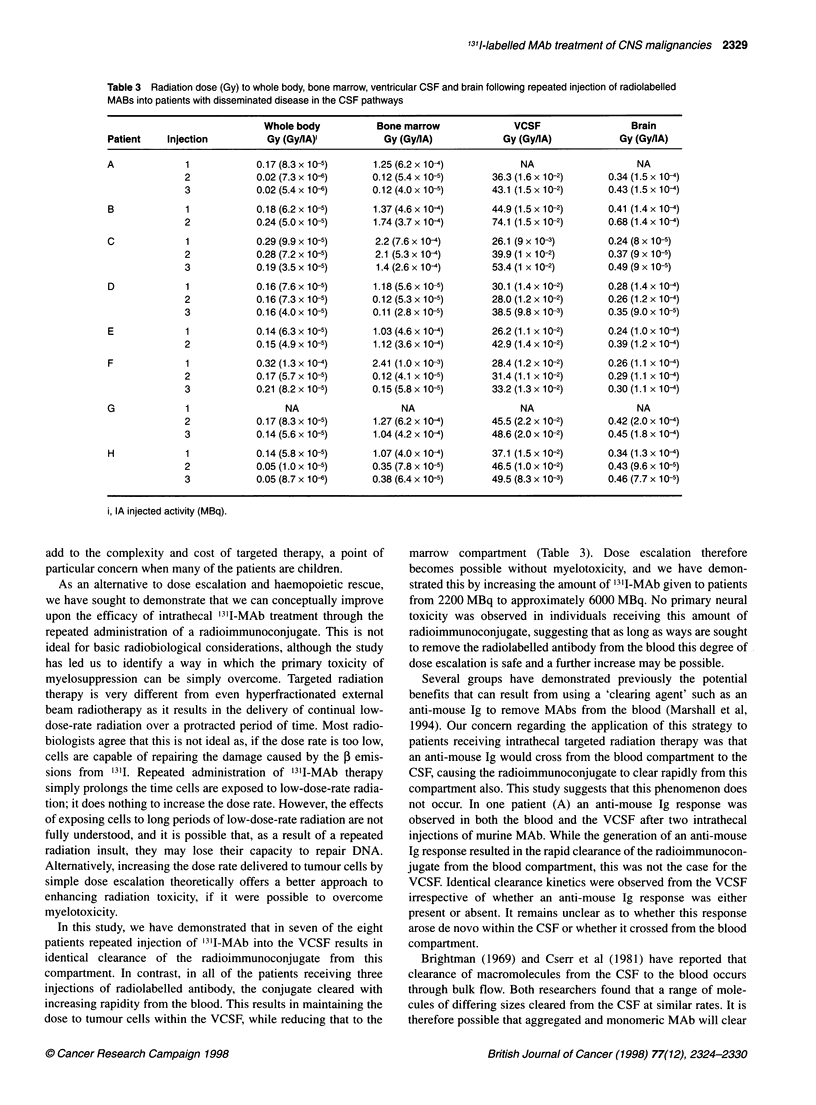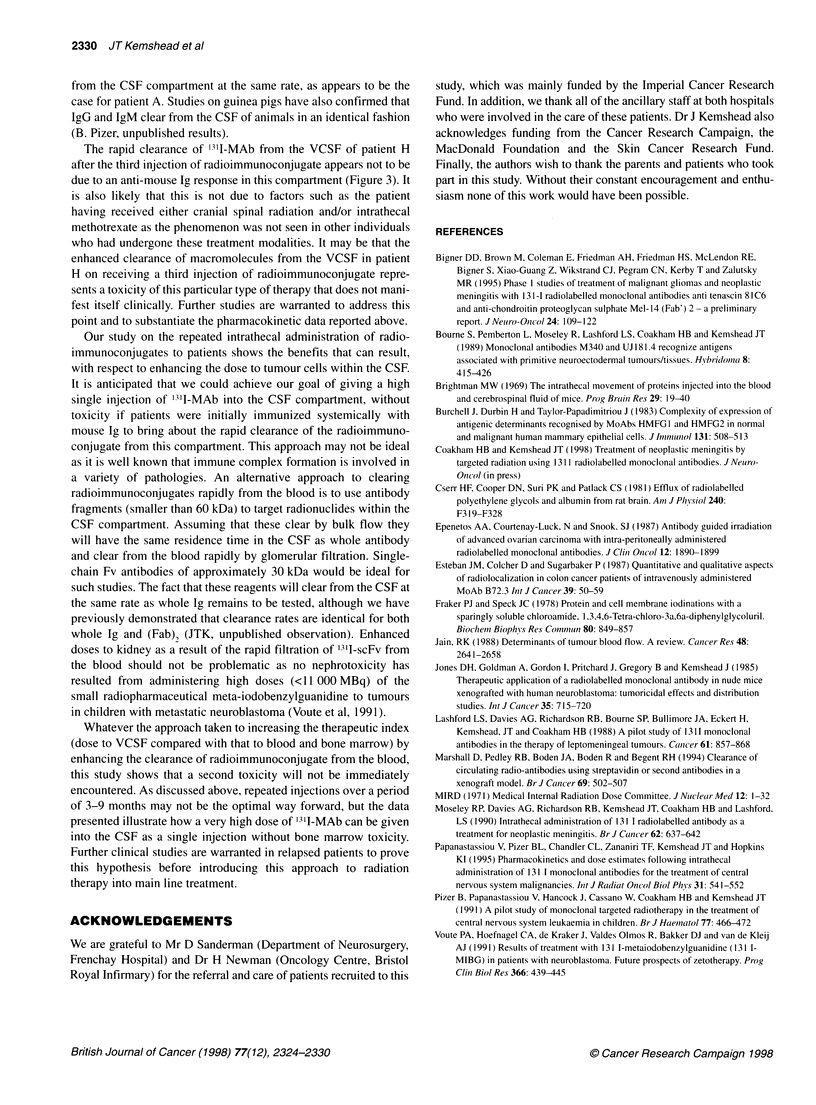# Dose escalation with repeated intrathecal injections of 131I-labelled MAbs for the treatment of central nervous system malignancies.

**DOI:** 10.1038/bjc.1998.386

**Published:** 1998-06

**Authors:** J. T. Kemshead, K. Hopkins, B. Pizer, V. Papanastassiou, H. Coakham, J. Bullimore, C. Chandler

**Affiliations:** Division of Oncology, University of Bristol, The Oncology Centre, UK.

## Abstract

We have previously demonstrated a 33% response rate in patients with primitive neurectodermal tumours after the direct injection of 131I-monoclonal antibodies (MAbs) into the cerebrospinal fluid (CSF). Dose-limiting toxicity is myelosuppression due to the passage of the radioimmunoconjugate from the CSF to the blood compartment. This occurs at doses of 2220 MBq of 131I-MAb and above, although this is not seen in all patients studied and appears to be related to the degree of prior therapy received. Rather than attempting to improve the efficacy of this approach to the treatment of disseminated disease within the CSF compartment by dose escalation and haemopoietic rescue, we have explored the possibility of repeatedly administering the radioimmunoconjugate. Eight patients were recruited to the study, two of whom received two and six of whom received three injections of 131I-MAb. After repeated administration of 131I-MAb pharmacokinetic data revealed that, with one exception, the radioimmunoconjugate cleared from the CSF compartment with similar kinetics, while its residence time in the blood decreased with each injection. This was due to the development of an anti-mouse Ig response in the blood. Clearance of 131I-MAb from the ventricular CSF appears to be independent of the presence of an anti-mouse Ig response in this compartment. The differential clearance of the radioimmunoconjugate from the ventricular CSF and from the blood results in a marked increase in the therapeutic index that can be achieved. Up to 5920 MBq of 131I-MAb was administered as the third injection of radioimmunoconjugate and combined doses of up to 12,500 MBq were given without either haematological or neurological toxicity. These data illustrate that dose escalation and thus an increase in the dose rate delivered to tumour cells within the CSF is possible if ways are found to reduce the residence time of the radioimmunoconjugate in the blood compartment. Suggestions as to how this can best be achieved are reviewed in detail.


British Journal of Cancer (1998) 77(12), 2324–2330
© 1998 Cancer Research Campaign

### Dose escalation with repeated intrathecal injections of $^{131}$I-labelled MAbs for the treatment of central nervous system malignancies

**JT Kemshead[1,2], K Hopkins[2], B Pizer[2], V Papanastassiou[2], H Coakham[3], J Bullimore[4] and C Chandler[3]**

[1]Division of Oncology, University of Bristol, The Oncology Centre, Horfield Road, Bristol BS2 8ED; [2]Paediatric and Neuro-Oncology Laboratories, Frenchay Hospital, Bristol BS16 1LE; [3]Department of Neurosurgery, Frenchay Hospital, Bristol BS16 1LE; [4]Division of Oncology, The Oncology Centre, Horfield Road, Bristol BS2 8ED, UK

**Summary** We have previously demonstrated a 33% response rate in patients with primitive neurectodermal tumours after the direct injection of $^{131}$I-monoclonal antibodies (MAbs) into the cerebrospinal fluid (CSF). Dose-limiting toxicity is myelosuppression due to the passage of the radioimmunoconjugate from the CSF to the blood compartment. This occurs at doses of 2220 MBq of $^{131}$I-MAb and above, although this is not seen in all patients studied and appears to be related to the degree of prior therapy received. Rather than attempting to improve the efficacy of this approach to the treatment of disseminated disease within the CSF compartment by dose escalation and haemopoietic rescue, we have explored the possibility of repeatedly administering the radioimmunoconjugate. Eight patients were recruited to the study, two of whom received two and six of whom received three injections of $^{131}$I-MAb. After repeated administration of $^{131}$I-MAb pharmacokinetic data revealed that, with one exception, the radioimmunoconjugate cleared from the CSF compartment with similar kinetics, while its residence time in the blood decreased with each injection. This was due to the development of an anti-mouse Ig response in the blood. Clearance of $^{131}$I-MAb from the ventricular CSF appears to be independent of the presence of an anti-mouse Ig response in this compartment. The differential clearance of the radioimmunoconjugate from the ventricular CSF and from the blood results in a marked increase in the therapeutic index that can be achieved. Up to 5920 MBq of $^{131}$I-MAb was administered as the third injection of radioimmunoconjugate and combined doses of up to 12 500 MBq were given without either haematological or neurological toxicity. These data illustrate that dose escalation and thus an increase in the dose rate delivered to tumour cells within the CSF is possible if ways are found to reduce the residence time of the radioimmunoconjugate in the blood compartment. Suggestions as to how this can best be achieved are reviewed in detail.

**Keywords:** monoclonal antibodies; central nervous system; malignancy; pharmacokinetics; dosimetry

Over the last decade it has become apparent that considerable problems exist in using monoclonal antibodies (MAbs) as delivery vehicles for anti-cancer agents in patients. The extremely encouraging responses seen in mice xenografted with human tumours have not been translated into the clinic (Jones et al, 1985). Low levels of antibody uptake into patient's solid tumour deposits have been observed compared with those seen in animal studies (Esteban et al, 1987). In addition, there is marked heterogeneity in the distribution of MAbs within solid tumours. These problems are thought to be brought about by the relatively high interstitial pressure in tumours compared with normal tissues, which limits the diffusion of large molecules, such as immunoglobulins (Jain, 1988). A further problem associated with the use of MAbs in patients compared with animals is the difference in the volume of distribution of the radioimmunoconjugate, potentially reducing the ability of MAbs to target to tumour. To attempt to overcome these problems several groups have concentrated their efforts on administering antibodies into body compartments that contain relatively small amounts of diffuse tumour (Epenetos et al, 1987; Lashford et al, 1988).

*Received 1 September 1997*
*Accepted 10 November 1997*

*Correspondence to:* J Kemshead, Paediatric and Neuro-Oncology Laboratories, Frenchay Hospital, Bristol BS16 1LE, UK

The cerebrospinal fluid (CSF) is conceptually ideal for targeting studies as this exists within a closed compartment of approximately 125 cm$^3$. It comprises the lateral cerebral ventricles, the third and fourth ventricles and the sub-arachnoid space. Cerebrospinal fluid is produced from the choroid plexus within the ventricles and this flows into the sub-arachnoid space through three foramina. CSF is produced at the rate of approximately 25 ml h$^{-1}$. To maintain a constant volume it is absorbed primarily through the arachnoid granulations, although it is also reported to enter the lymphatics and leave the compartment through leakage from the nerve roots.

We have previously reported a 33% response rate in patients with disseminated primitive neuroectodermal tumours (PNET) after a single bolus intraventricular injection of $^{131}$I-MAbs into their CSF, through an Ommaya reservoir (Coakham and Kemshead, 1998). Patients were selected for study based upon their having either diffuse disseminated disease and/or small tumour nodules no larger than 1.5 cm in diameter, and no blockage in their CSF pathways. As part of these Phase I studies, we identified the primary toxicity of this type of therapy as myelosuppression, which occurred idiosyncratically at doses of 2220 MBq of $^{131}$I-MAb and above (Papanastassiou et al, 1995). Pharmacokinetic data indicate that myelosuppression is brought about through the passage of the radioimmunoconjugate from the CSF into the blood compartment. This clearly limits the possibility of increasing the efficacy of targeting by simple dose escalation.

**Table 1** Details of patients recruited to the study

| Patient | Age (years) | Diagnosis | MAb | Number of injections | Duration of treatment (months) |
|---|---|---|---|---|---|
| A | 7 | PNET (medulloblastoma) | M340[a] | 3 | 9 |
| B | 37 | Carcinomatous meningitis (breast) | HMFG1[b] | 2 | 1 |
| C | 27 | PNET (medulloblastoma) | M340 | 3 | 5 |
| D | 3 | Acute lymphoblastic leukaemia | WCMH[c] | 3 | 9 |
| E | 4 | PNET (medulloblastoma) | M340 | 2 | 3 |
| F | 43 | PNET (medulloblastoma) | M340 | 3 | 9 |
| G | 9 | PNET (ependymoblastoma) | M340 | 3 | 9 |
| H | 14 | PNET (pineoblastoma) | M340 | 3 | 3 |

[a]Bourne et al (1989). [b]Burchell et al (1983). [c]Pizer et al (1991).

**Table 2** Area under the time activity curves for ventricular CSF and blood following repeated injection of radiolabelled MABs into patients with disseminated disease in the CSF pathways

| Patient | Injection | IA (MBq)[a] | AUCVCSF[b] | AUC VCSF/IA[c] | AUCBL[d] | AUCBL/IA[e] | AUCVCS/ AVCBL[f] |
|---|---|---|---|---|---|---|---|
| A | 1 | 2026 | NA | – | 15.99 | $7.8 \times 10^{-3}$ | NA |
| | 2 | 2267 | 294 | 0.12 | 1.58 | $6.9 \times 10^{-4}$ | 186 |
| | 3 | 2917 | 368 | 0.12 | 1.52 | $5.2 \times 10^{-4}$ | 242 |
| B | 1 | 2959 | 363 | 0.12 | 15.5 | $5.2 \times 10^{-3}$ | 23.4 |
| | 2 | 4712 | 599 | 0.12 | 19.7 | $4.1 \times 10^{-3}$ | 30.4 |
| C | 1 | 2856 | 211 | 0.07 | 15.7 | $5.4 \times 10^{-3}$ | 13.4 |
| | 2 | 3869 | 323 | 0.08 | 15.2 | $3.9 \times 10^{-3}$ | 21.2 |
| | 3 | 5362 | 432 | 0.08 | 10.3 | $1.9 \times 10^{-3}$ | 41.9 |
| D | 1 | 2101 | 244 | 0.11 | 28.5 | $1.3 \times 10^{-2}$ | 8.5 |
| | 2 | 2236 | 227 | 0.10 | 2.96 | $1.3 \times 10^{-3}$ | 76.6 |
| | 3 | 3920 | 343 | 0.09 | 2.67 | $6.8 \times 10^{-4}$ | 128.4 |
| E | 1 | 2205 | 212 | 0.09 | 37.2 | $1.6 \times 10^{-2}$ | 5.6 |
| | 2 | 3050 | 347 | 0.11 | 40.3 | $1.3 \times 10^{-2}$ | 8.1 |
| F | 1 | 2380 | 230 | 0.09 | 18.4 | $8.5 \times 10^{-3}$ | 12.5 |
| | 2 | 2917 | 254 | 0.08 | 1.50 | $5.4 \times 10^{-4}$ | 169.3 |
| | 3 | 2553 | 269 | 0.11 | 1.88 | $7.3 \times 10^{-4}$ | 196.2 |
| G | 1 | 2142 | NA | – | NA | – | NA |
| | 2 | 2035 | 368 | 0.18 | 7.70 | $3.7 \times 10^{-3}$ | 47.7 |
| | 3 | 2460 | 433 | 0.18 | 5.14 | $2.0 \times 10^{-3}$ | 84.2 |
| H | 1 | 2470 | 300 | 0.12 | 14.38 | $5.8 \times 10^{-3}$ | 20.8 |
| | 2 | 4446 | 376 | 0.09 | 4.63 | $1.0 \times 10^{-3}$ | 81.2 |
| | 3 | 5920 | 400 | 0.07 | 4.92 | $5.5 \times 10^{-4}$ | 81.3 |

[a]Injected activity into the ventricular CSF in MBq. [b]Area under the VCSF time activity curve MBq h $ml^{-1}$. [c]Area under the VCSF time activity curve/MBq administered MBq h $ml^{-1}$. [d]Area under the blood time activity curve MBq h $ml^{-1}$. [e]Area under the VCSF time activity curve/MBq administered MBq h $ml^{-1}$. [f]Area under the VCSF time activity curve/area under the blood time activity curve.

To enhance the response of patients to targeted radiation therapy, we have sought to administer repeated injections of radioimmunoconjugate to a number of individuals with diffuse malignant infiltrates within the CSF compartment. Pharmacokinetic studies indicate that, in general, the clearance kinetics of the radioimmunoconjugate from the ventricular CSF (VCSF) remain constant throughout the course of antibody therapy. This contrasts to the increasingly rapid removal of material from the blood, brought about by the induction of an anti-mouse Ig response. These findings have enabled us to escalate markedly the amount of $^{131}$I-MAb given to patients after their primary injection. Within this context, no haematological or other toxicity was encountered in patients receiving either a single intrathecal injection of up to 5920 MBq of $^{131}$I-MAb or a cumulative dose of radioimmunoconjugate in excess of 12 500 MBq given over a 3-month period. Based upon this data, we propose that it should be possible to overcome the primary toxicity of targeted radiation therapy within the CSF compartment, and we review ways in which this goal can be achieved.

### MATERIALS AND METHODS

### Patient recruitment

Ethical committee approval for this study was obtained from the relevant committees at Frenchay Hospital and the Bristol Royal Infirmary. Patients were recruited to the study after failure of conventional treatment. All had diffuse leptomeningeal deposits

© Cancer Research Campaign 1998

without evidence of either solid parenchymal disease or spinal block. Before treatment patients had an Ommaya reservoir inserted into a lateral ventricle to facilitate the administration of $^{131}$I-MAbs and to allow ventricular CSF sampling.

Informed consent was obtained before the treatment and patients were placed on a regimen to prevent free iodine accumulation in the thyroid gland. Patients were also prescribed dexamethasone and phenytoin to reduce the risks of cerebral oedema and fits respectively (Papanastassiou et al, 1995).

### Monoclonal antibodies

Antibodies were chosen on the basis of their binding to either frozen sections of patient's tumour or malignant cells within the CSF (Table 1). These reagents have also been screened extensively to demonstrate that they do not cross-react with normal neural elements. Monoclonal antibodies were conjugated to $^{131}$I using the iodogen technique to a specific activity of approximately 370 MBq mg$^{-1}$ (Fraker and Speck, 1978). After radiolabelling, conjugates were screened for the presence of free iodine by trichloroacetic acid precipitation and fast protein liquid chromatography (FPLC), for the presence of aggregates by FPLC and for immunoreactivity. In addition, samples were assayed for endotoxins (limulus test) and for sterility. Radiolabelled MAbs were administered into the patient's Ommaya reservoir as a bolus injection through a 0.22-µm filter. Radioimmunoconjugates were injected within 6 h of preparation to avoid the possibility of radiolysis.

It was our intention to administer three courses of radioimmunotherapy to each patient, at approximately 4-week intervals. For a variety of clinical and logistical reasons, this schedule was not maintained. Brief details of the patients entered into the study are presented in Table 1.

**Figure 1** Effective clearance of $^{131}$I-MAb from the ventricular CSF of patient C. Samples were taken from the Ommaya reservoir at different times and the isotope levels determined in a LKB compugamma counter. ■, Clearance curve after the first injection of radioimmunoconjugate; □, clearance curve after the second injection of radioimmunoconjugate; ▲, clearance curve after the third injection of radioimmunoconjugate

### Patient sampling

Samples were taken at frequent intervals to determine the clearance of radioimmunoconjugate from the blood and VCSF. These were allowed to decay for a known period so that it was possible to determine the level of radioactivity per unit volume in a LKB Ultra-gamma counter. Knowing the efficiency of the counter, the time of counting in relation to the time of immunoconjugate administration and the decay constant of the isotope, the biological and effective clearance curves for the isotope from VCSF and blood could be calculated. The areas under the effective clearance curves for VCSF and blood were determined from $T_0$ until the last data point (approximately 168 h) by the linear-trapezoid rule. The areas under the curves from the last data point to infinity were calculated by integration. The total areas under these curves represent the time activity integrals for blood and CSF.

### Dosimetry

To calculate the radiation dose to whole body, bone marrow, VCSF and whole brain, the medical internal radiation dose (MIRD) formalism was used (MIRD, 1971). A detailed description of the methods used to determine the dose to these organs as a result of intrathecal targeted radiation has been described previously (Papanastassiou et al, 1995).

### RESULTS

### Patients and $^{131}$I-MAb administration

In total, eight patients were given repeated injections of $^{131}$I-MAbs, two of these receiving two injections and the remainder three. Patients received their targeted therapy over periods ranging from 1 to 9 months (Table 1). The six patients with PNETs received multiple injections of radiolabelled MAb M340, while the other two received either HMFG1 (patient B with carcinomatous meningitis arising from a breast primary) or the anti-CD10 MAb WCMH (patient D). Five of the eight patients were children (A, D, E, G, H) and all of these presented with PNETs apart from D who was diagnosed with common acute lymphoblastic leukaemia (c-ALL). Patients received between 2026 and 2959 MBq of $^{131}$I-MAbs as their initial injection, and this was dose escalated to a maximum of 5920 MBq (patient H, Table 2). The total amount of radioimmunoconjugate given to patients ranged from 5255 to 12 836 MBq (patients E and H respectively; Table 2).

In all instances the specific activity of the radioimmunoconjugate remained constant (approximately 10 µCi µg$^{-1}$). Preparations contained less than 5% aggregates and 5% free iodine. The immunoreactivity of the samples varied from 60% to 80%.

### Toxicity

### *Acute*

The acute toxicity observed in this study mirrored that previously reported for patients receiving intrathecal targeted radiation therapy (Papanastassiou et al, 1995). After the first injection of radioimmunoconjugate, an acute aseptic meningitis was observed in half of the patients (D, E, F, G) which resolved within 72 h. When these patients received their second and subsequent injections of $^{131}$I-MAb, a transient aseptic meningitis reoccurred. No acute toxicity was observed in the other half of the group, apart

 © Cancer Research Campaign 1998

from in patient B. In this individual, the second injection of $^{131}$I-MAb caused an elevation of intracranial pressure, which was controlled by aspiration and the use of intravenous mannitol.

### *Dose-limiting toxicity*

World Health Organization (WHO) grade 3/4 myelosuppression was observed in three of the eight patients. In patient A, this occurred 3 weeks after the first injection of 2026 MBq of radioimmunoconjugate. Subsequent injections of 2267 and 2917 MBq of $^{131}$I MAb did not cause detectable myelotoxicity. Patient D, who was heavily pretreated with combination chemotherapy, continued on systemic maintenance chemotherapy for c-ALL throughout his course of targeted radiation therapy. Myelosuppression occurred as a result of each injection of radioimmunoconjugate, this being treated on each occasion with granulocyte–macrophage colony-stimulating factor (GM-CSF). It is therefore not possible to comment accurately on the degree of myelosuppression caused as a consequence of the radiolabelled antibody. Patient E only received two injections of $^{131}$I-M340, the second being given while she was pancytopenic. During this period, she also received GM-CSF.

The maximum single dose of $^{131}$I-M340 administered to patients was 5920 MBq. This was given as the third course of therapy to patient H. No neural or haemopoietic toxicity was encountered as a result of this injection. Several other patients also received, as either their second or third injections of $^{131}$I-MAb, doses of radioimmunoconjugate in excess of 3000 MBq, without either haematological or neural toxicity. Analysis of the pharmacokinetic data presented below illustrates the reasons underlying the lack of myelotoxicity and suggests that considerable dose escalation is possible.

### Pharmacokinetics

Over a period of 7 days, samples of ventricular CSF and blood were taken from all of the patients. Wherever possible at least four samples were acquired within the first 24 h, two within the second

**Figure 2** Effective clearance of $^{131}$I-MAb from the blood of patient C. Samples (1–2 ml) were taken at different times and the isotope levels determined in a LKB compugamma counter. ■, Clearance curve after the first injection of radioimmunoconjugate; □, clearance curve after the second injection of radioimmunoconjugate; ▲, clearance curve after the third injection of radioimmunoconjugate day after MAb administration and daily thereafter. From this data, effective clearance curves were constructed for both ventricular CSF and the blood. Figure 1 illustrates the data obtained from patient C, expressed as the percentage of injected dose administered. The VCSF clearance curves for this individual were typical of the whole population studied. He received three injections of $^{131}$I-M340 of 2856, 3869 and 5362 MBq. Clearance of $^{131}$I-MAb from the VCSF followed biphasic clearance kinetics and remained constant for all three injections of radioimmunoconjugate. In contrast, the percentage of the injected dose of radioimmunoconjugate in the blood fell after each injection of radiolabelled MAb. Peak blood levels reached 42% at 32 h after the first injection of radioimmunoconjugate, falling to just over 30% and 20% for the second and third injections (Figure 2).

For patient C, the time activity integrals for the clearance of $^{131}$I-M340 from VCSF were calculated as 211, 323 and 432 MBq h ml$^{-1}$ for the three injections (Table 2). This increase reflects the amount of radioimmunoconjugate administered, which was almost doubled over the course of treatment. However, when this data is normalized for the quantity of radioimmunoconjugate administered, the time activity integral/unit injected activity remains constant for each injection (0.07, 0.08 and 0.08 respectively).

A similar analysis of the blood clearance data for patient C reveals time activity integrals of 15.7, 15.2 and 10.3 MBq h ml$^{-1}$ for injections one, two and three. Thus, in contrast to the VCSF data, the time activity integral for the radioimmunoconjugate in the blood falls despite an increase in the amount of radionuclide administered. This leads to a marked increase in the ratio of the area under the VCSF–blood time activity curves (AUCVCSF/AUCBlood). For patient C, this ratio rose from 13.4 after the first injection of radioimmunoconjugate to 41.9 after the third injection (Table 2).

It was not possible to obtain complete data sets for all eight patients for logistical reasons. However, in all cases, apart from patient H, the ratio of the area under the VCSF–blood time activity curves increased after each injection of radioimmunoconjugate. After the third injection of $^{131}$I MAb to patient H, the ratio of AUCVCSF/AUCBlood was found to be identical to that after the second injection (Table 2). This was due to a marked increase in the clearance rate of the $^{131}$I-MAb from the VCSF of this patient after his third injection of $^{131}$I-MAb.

In four of the eight patients, the increase in the AUCVCSF/AUCblood ratio was mainly brought about by a significant decrease in the blood time activity integral after repeated injections of $^{131}$I-MAb. In two of the other three (B and E), the time activity integral in blood increased as the dose administered to the VCSF was escalated. However, in these patients an increase in the ratio between time activity integral in the VCSF–blood was still observed, reflecting the differential clearance of the radioimmunoconjugate from the two body compartments. In the case of patients A and G, full data were only available after the first injection of $^{131}$I-MAb, making a complete analysis impossible.

### Anti-mouse Ig responses in blood and CSF

CSF and serum samples were acquired before the start of the study from three patients (A, C and H). These were mixed in vitro with $^{131}$I-MAb to determine whether they affected the monomeric status of the radiolabelled antibody. In all cases, FPLC analysis revealed that the antibody alone resolved as a single band of approximately 150 kDa, and identical traces were obtained after preincubation of

**Figure 3** Fast protein liquid chromatography of $^{131}$I-MAb samples after mixing with either blood or CSF samples. (**A**) Serum and CSF samples taken from patient C before the MAb study. (**B**) Serum and CSF samples taken from patient C before the third injection of MAb. (**C**) Serum and CSF samples taken from patient A before the third injection of MAb. ◇, $^{131}$I-MAb diluted in phosphate-buffered saline; □, $^{131}$I-MAb mixed with serum; ○, $^{131}$I-MAb mixed with CSF

the reagent with either serum or CSF (Figure 3A). This study was repeated with samples acquired before administration of the third injection of radioimmunoconjugate. When these were mixed with $^{131}$I-MAb, radionuclide in the serum sample was associated with a high-molecular-weight peak, indicating that the immunoglobulin had aggregated, presumably as a result of the generation of an anti-mouse Ig response (Figure 3B). No aggregation of $^{131}$I-M340 was seen in two of the three CSF samples taken before the third injection of radioimmunoconjugate (Patients C and H). In the other VCSF sample from patient A, marked aggregation occurred (Figure 3C). A semiquantitative radioimmune assay for the presence of an anti-mouse Ig response in blood and CSF confirmed the above findings, indicating that an anti-mouse Ig response was detected in only the VCSF sample taken from patient A before his third injection of radiolabelled MAb (data not presented).

### Dosimetry

Repeated injections of increasing amounts of $^{131}$I-MAbs resulted in a marked rise in the calculated radiation dose to the VCSF, which is representative of that received by free-floating tumour cells within the fluid. For example, in patient C, the dose to the VCSF increased from 26 Gy as a consequence of the first injection to 53 Gy after the third (Table 3). In contrast, the bone marrow dose in this patient fell from 1.9 to 1.4 Gy. Considerably larger decreases in the bone marrow dose were observed in other individuals, ranging from three- to 20-fold. These occurred despite a marked increase in radionuclide being administered to patients (Tables 2 and 3). Whole-body doses remained low throughout the study, these also falling as a consequence of the radioimmunoconjugate clearing with increasing rapidity from the blood. The dose to whole brain remained acceptable throughout the study, ranging from 0.24 to 0.68 Gy. The dose to this organ either remained constant or fell slightly as the amount of radioimmunoconjugate was increased throughout each patient's treatment.

### Response

Because of the size of the group studied, it is clearly not possible to determine whether repeating targeted radiation therapy in the CSF compartment enhances the efficacy of treatment compared with the results observed after a single bolus injection. However, for completeness, the clinical outcome of the patients entered into the study is documented.

In the PNET group, responses were observed in four of the six individuals. Patient C remains alive and disease free 46 months from the completion of treatment. A complete clearance of cells from the CSF for 1 month after both the first and the second injections of $^{131}$I-MAb was observed in patient E, who subsequently deteriorated rapidly. Patient F had a complete response (CR) for 4 months after the first injection of radioimmunoconjugate. He was then given two further injections 1 month apart and a complete clearance of cells from the CSF was observed for a further 8 months. Finally, patient H received three injections of $^{131}$I-M340 at monthly intervals. He had a complete response for 10 months before rapidly relapsing with extensive disease. Patient A remained with static disease for 12 months before relapse, and patient G was not evaluable for response as he was treated after surgery, in the adjuvant setting. He currently remains disease free 24 months from treatment.

Patient B with carcinomatous meningitis had a partial response to antibody therapy, clearing cells from the CSF for periods of 1 and 2 months after each injection of $^{131}$I-MAb. She subsequently relapsed and was considered too ill to receive further targeted therapy. Patient D was not possible to evaluate for response as he was also treated in the adjuvant setting. However, the period of his third remission exceeded that of the second, suggesting that he may have received some benefit from the radioimmunotherapy.

### DISCUSSION

Previously, both our group and others identified the primary toxicity of intrathecal targeted radiation therapy as myelosuppression, brought about by the clearance of the radiolabelled MAb from the CSF compartment to the blood. Dose-limiting toxicity occurs at an activity of approximately 2220 MBq of $^{131}$I-MAb, although this varies from patient to patient (Moseley et al, 1989; Bigner et al, 1995; Papanastassiou et al, 1995). The amount of combination chemotherapy given to individuals before targeted radiation therapy tends to render them more susceptible to myelosuppression. It is clearly possible to dose escalate above this toxicity, through the use of either an autologous or allogeneic bone marrow rescue procedure, as long as another toxicity does not immediately become dose limiting. However, both of these techniques greatly

 © Cancer Research Campaign 1998

**Table 3** Radiation dose (Gy) to whole body, bone marrow, ventricular CSF and brain following repeated injection of radiolabelled MABs into patients with disseminated disease in the CSF pathways

| Patient | Injection | Whole body Gy (Gy/IA)[i] | Bone marrow Gy (Gy/IA) | VCSF Gy (Gy/IA) | Brain Gy (Gy/IA) |
|---|---|---|---|---|---|
| A | 1 | 0.17 ($8.3 \times 10^{-5}$) | 1.25 ($6.2 \times 10^{-4}$) | NA | NA |
| | 2 | 0.02 ($7.3 \times 10^{-6}$) | 0.12 ($5.4 \times 10^{-5}$) | 36.3 ($1.6 \times 10^{-2}$) | 0.34 ($1.5 \times 10^{-4}$) |
| | 3 | 0.02 ($5.4 \times 10^{-6}$) | 0.12 ($4.0 \times 10^{-5}$) | 43.1 ($1.5 \times 10^{-2}$) | 0.43 ($1.5 \times 10^{-4}$) |
| B | 1 | 0.18 ($6.2 \times 10^{-5}$) | 1.37 ($4.6 \times 10^{-4}$) | 44.9 ($1.5 \times 10^{-2}$) | 0.41 ($1.4 \times 10^{-4}$) |
| | 2 | 0.24 ($5.0 \times 10^{-5}$) | 1.74 ($3.7 \times 10^{-4}$) | 74.1 ($1.5 \times 10^{-2}$) | 0.68 ($1.4 \times 10^{-4}$) |
| C | 1 | 0.29 ($9.9 \times 10^{-5}$) | 2.2 ($7.6 \times 10^{-4}$) | 26.1 ($9 \times 10^{-3}$) | 0.24 ($8 \times 10^{-5}$) |
| | 2 | 0.28 ($7.2 \times 10^{-5}$) | 2.1 ($5.3 \times 10^{-4}$) | 39.9 ($1 \times 10^{-2}$) | 0.37 ($9 \times 10^{-5}$) |
| | 3 | 0.19 ($3.5 \times 10^{-5}$) | 1.4 ($2.6 \times 10^{-4}$) | 53.4 ($1 \times 10^{-2}$) | 0.49 ($9 \times 10^{-5}$) |
| D | 1 | 0.16 ($7.6 \times 10^{-5}$) | 1.18 ($5.6 \times 10^{-5}$) | 30.1 ($1.4 \times 10^{-2}$) | 0.28 ($1.4 \times 10^{-4}$) |
| | 2 | 0.16 ($7.3 \times 10^{-5}$) | 0.12 ($5.3 \times 10^{-5}$) | 28.0 ($1.2 \times 10^{-2}$) | 0.26 ($1.2 \times 10^{-4}$) |
| | 3 | 0.16 ($4.0 \times 10^{-5}$) | 0.11 ($2.8 \times 10^{-5}$) | 38.5 ($9.8 \times 10^{-3}$) | 0.35 ($9.0 \times 10^{-5}$) |
| E | 1 | 0.14 ($6.3 \times 10^{-5}$) | 1.03 ($4.6 \times 10^{-4}$) | 26.2 ($1.1 \times 10^{-2}$) | 0.24 ($1.0 \times 10^{-4}$) |
| | 2 | 0.15 ($4.9 \times 10^{-5}$) | 1.12 ($3.6 \times 10^{-4}$) | 42.9 ($1.4 \times 10^{-2}$) | 0.39 ($1.2 \times 10^{-4}$) |
| F | 1 | 0.32 ($1.3 \times 10^{-4}$) | 2.41 ($1.0 \times 10^{-3}$) | 28.4 ($1.2 \times 10^{-2}$) | 0.26 ($1.1 \times 10^{-4}$) |
| | 2 | 0.17 ($5.7 \times 10^{-5}$) | 0.12 ($4.1 \times 10^{-5}$) | 31.4 ($1.1 \times 10^{-2}$) | 0.29 ($1.1 \times 10^{-4}$) |
| | 3 | 0.21 ($8.2 \times 10^{-5}$) | 0.15 ($5.8 \times 10^{-5}$) | 33.2 ($1.3 \times 10^{-2}$) | 0.30 ($1.1 \times 10^{-4}$) |
| G | 1 | NA | NA | NA | NA |
| | 2 | 0.17 ($8.3 \times 10^{-5}$) | 1.27 ($6.2 \times 10^{-4}$) | 45.5 ($2.2 \times 10^{-2}$) | 0.42 ($2.0 \times 10^{-4}$) |
| | 3 | 0.14 ($5.6 \times 10^{-5}$) | 1.04 ($4.2 \times 10^{-4}$) | 48.6 ($2.0 \times 10^{-2}$) | 0.45 ($1.8 \times 10^{-4}$) |
| H | 1 | 0.14 ($5.8 \times 10^{-5}$) | 1.07 ($4.0 \times 10^{-4}$) | 37.1 ($1.5 \times 10^{-2}$) | 0.34 ($1.3 \times 10^{-4}$) |
| | 2 | 0.05 ($1.0 \times 10^{-5}$) | 0.35 ($7.8 \times 10^{-5}$) | 46.5 ($1.0 \times 10^{-2}$) | 0.43 ($9.6 \times 10^{-5}$) |
| | 3 | 0.05 ($8.7 \times 10^{-6}$) | 0.38 ($6.4 \times 10^{-5}$) | 49.5 ($8.3 \times 10^{-3}$) | 0.46 ($7.7 \times 10^{-5}$) |

i, IA injected activity (MBq).

add to the complexity and cost of targeted therapy, a point of particular concern when many of the patients are children.

As an alternative to dose escalation and haemopoietic rescue, we have sought to demonstrate that we can conceptually improve upon the efficacy of intrathecal $^{131}$I-MAb treatment through the repeated administration of a radioimmunoconjugate. This is not ideal for basic radiobiological considerations, although the study has led us to identify a way in which the primary toxicity of myelosuppression can be simply overcome. Targeted radiation therapy is very different from even hyperfractionated external beam radiotherapy as it results in the delivery of continual low-dose-rate radiation over a protracted period of time. Most radiobiologists agree that this is not ideal as, if the dose rate is too low, cells are capable of repairing the damage caused by the β emissions from $^{131}$I. Repeated administration of $^{131}$I-MAb therapy simply prolongs the time cells are exposed to low-dose-rate radiation; it does nothing to increase the dose rate. However, the effects of exposing cells to long periods of low-dose-rate radiation are not fully understood, and it is possible that, as a result of a repeated radiation insult, they may lose their capacity to repair DNA. Alternatively, increasing the dose rate delivered to tumour cells by simple dose escalation theoretically offers a better approach to enhancing radiation toxicity, if it were possible to overcome myelotoxicity.

In this study, we have demonstrated that in seven of the eight patients repeated injection of $^{131}$I-MAb into the VCSF results in identical clearance of the radioimmunoconjugate from this compartment. In contrast, in all of the patients receiving three injections of radiolabelled antibody, the conjugate cleared with increasing rapidity from the blood. This results in maintaining the dose to tumour cells within the VCSF, while reducing that to the marrow compartment (Table 3). Dose escalation therefore becomes possible without myelotoxicity, and we have demonstrated this by increasing the amount of $^{131}$I-MAb given to patients from 2200 MBq to approximately 6000 MBq. No primary neural toxicity was observed in individuals receiving this amount of radioimmunoconjugate, suggesting that as long as ways are sought to remove the radiolabelled antibody from the blood this degree of dose escalation is safe and a further increase may be possible.

Several groups have demonstrated previously the potential benefits that can result from using a 'clearing agent' such as an anti-mouse Ig to remove MAbs from the blood (Marshall et al, 1994). Our concern regarding the application of this strategy to patients receiving intrathecal targeted radiation therapy was that an anti-mouse Ig would cross from the blood compartment to the CSF, causing the radioimmunoconjugate to clear rapidly from this compartment also. This study suggests that this phenomenon does not occur. In one patient (A) an anti-mouse Ig response was observed in both the blood and the VCSF after two intrathecal injections of murine MAb. While the generation of an anti-mouse Ig response resulted in the rapid clearance of the radioimmunoconjugate from the blood compartment, this was not the case for the VCSF. Identical clearance kinetics were observed from the VCSF irrespective of whether an anti-mouse Ig response was either present or absent. It remains unclear as to whether this response arose de novo within the CSF or whether it crossed from the blood compartment.

Brightman (1969) and Cserr et al (1981) have reported that clearance of macromolecules from the CSF to the blood occurs through bulk flow. Both researchers found that a range of molecules of differing sizes cleared from the CSF at similar rates. It is therefore possible that aggregated and monomeric MAb will clear

© Cancer Research Campaign 1998 

from the CSF compartment at the same rate, as appears to be the case for patient A. Studies on guinea pigs have also confirmed that IgG and IgM clear from the CSF of animals in an identical fashion (B. Pizer, unpublished results).

The rapid clearance of $^{131}$I-MAb from the VCSF of patient H after the third injection of radioimmunoconjugate appears not to be due to an anti-mouse Ig response in this compartment (Figure 3). It is also likely that this is not due to factors such as the patient having received either cranial spinal radiation and/or intrathecal methotrexate as the phenomenon was not seen in other individuals who had undergone these treatment modalities. It may be that the enhanced clearance of macromolecules from the VCSF in patient H on receiving a third injection of radioimmunoconjugate represents a toxicity of this particular type of therapy that does not manifest itself clinically. Further studies are warranted to address this point and to substantiate the pharmacokinetic data reported above.

Our study on the repeated intrathecal administration of radioimmunoconjugates to patients shows the benefits that can result, with respect to enhancing the dose to tumour cells within the CSF. It is anticipated that we could achieve our goal of giving a high single injection of $^{131}$I-MAb into the CSF compartment, without toxicity if patients were initially immunized systemically with mouse Ig to bring about the rapid clearance of the radioimmunoconjugate from this compartment. This approach may not be ideal as it is well known that immune complex formation is involved in a variety of pathologies. An alternative approach to clearing radioimmunoconjugates rapidly from the blood is to use antibody fragments (smaller than 60 kDa) to target radionuclides within the CSF compartment. Assuming that these clear by bulk flow they will have the same residence time in the CSF as whole antibody and clear from the blood rapidly by glomerular filtration. Single-chain Fv antibodies of approximately 30 kDa would be ideal for such studies. The fact that these reagents will clear from the CSF at the same rate as whole Ig remains to be tested, although we have previously demonstrated that clearance rates are identical for both whole Ig and $(Fab)_2$ (JTK, unpublished observation). Enhanced doses to kidney as a result of the rapid filtration of $^{131}$I-scFv from the blood should not be problematic as no nephrotoxicity has resulted from administering high doses (<11 000 MBq) of the small radiopharmaceutical meta-iodobenzylguanidine to tumours in children with metastatic neuroblastoma (Voute et al, 1991).

Whatever the approach taken to increasing the therapeutic index (dose to VCSF compared with that to blood and bone marrow) by enhancing the clearance of radioimmunoconjugate from the blood, this study shows that a second toxicity will not be immediately encountered. As discussed above, repeated injections over a period of 3–9 months may not be the optimal way forward, but the data presented illustrate how a very high dose of $^{131}$I-MAb can be given into the CSF as a single injection without bone marrow toxicity. Further clinical studies are warranted in relapsed patients to prove this hypothesis before introducing this approach to radiation therapy into main line treatment.

### ACKNOWLEDGEMENTS

We are grateful to Mr D Sanderman (Department of Neurosurgery, Frenchay Hospital) and Dr H Newman (Oncology Centre, Bristol Royal Infirmary) for the referral and care of patients recruited to this study, which was mainly funded by the Imperial Cancer Research Fund. In addition, we thank all of the ancillary staff at both hospitals who were involved in the care of these patients. Dr J Kemshead also acknowledges funding from the Cancer Research Campaign, the MacDonald Foundation and the Skin Cancer Research Fund. Finally, the authors wish to thank the parents and patients who took part in this study. Without their constant encouragement and enthusiasm none of this work would have been possible.

### REFERENCES

Bigner DD, Brown M, Coleman E, Friedman AH, Friedman HS, McLendon RE, Bigner S, Xiao-Guang Z, Wikstrand CJ, Pegram CN, Kerby T and Zalutsky MR (1995) Phase 1 studies of treatment of malignant gliomas and neoplastic meningitis with 131-I radiolabelled monoclonal antibodies anti tenascin 81C6 and anti-chondroitin proteoglycan sulphate Mel-14 (Fab') 2 – a preliminary report. *J Neuro-Oncol* **24**: 109–122

Bourne S, Pemberton L, Moseley R, Lashford LS, Coakham HB and Kemshead JT (1989) Monoclonal antibodies M340 and UJ181.4 recognize antigens associated with primitive neuroectodermal tumours/tissues. *Hybridoma* **8**: 415–426

Brightman MW (1969) The intrathecal movement of proteins injected into the blood and cerebrospinal fluid of mice. *Prog Brain Res* **29**: 19–40

Burchell J, Durbin H and Taylor-Papadimitriou J (1983) Complexity of expression of antigenic determinants recognised by MoAbs HMFG1 and HMFG2 in normal and malignant human mammary epithelial cells. *J Immunol* **131**: 508–513

Coakham HB and Kemshead JT (1998) Treatment of neoplastic meningitis by targeted radiation using 131I radiolabelled monoclonal antibodies. *J Neuro-Oncol* (in press)

Cserr HF, Cooper DN, Suri PK and Patlack CS (1981) Efflux of radiolabelled polyethylene glycols and albumin from rat brain. *Am J Physiol* **240**: F319–F328

Epenetos AA, Courtenay-Luck, N and Snook, SJ (1987) Antibody guided irradiation of advanced ovarian carcinoma with intra-peritoneally administered radiolabelled monoclonal antibodies. *J Clin Oncol* **12**: 1890–1899

Esteban JM, Colcher D and Sugarbaker P (1987) Quantitative and qualitative aspects of radiolocalization in colon cancer patients of intravenously administered MoAb B72.3 *Int J Cancer* **39**: 50–59

Fraker PJ and Speck JC (1978) Protein and cell membrane iodinations with a sparingly soluble chloroamide, 1,3,4,6-Tetra-chloro-3a,6a-diphenylglycoluril. *Biochem Biophys Res Commun* **80**: 849–857

Jain, RK (1988) Determinants of tumour blood flow. A review. *Cancer Res* **48**: 2641–2658

Jones DH, Goldman A, Gordon I, Pritchard J, Gregory B and Kemshead J (1985) Therapeutic application of a radiolabelled monoclonal antibody in nude mice xenografted with human neuroblastoma: tumoricidal effects and distribution studies. *Int J Cancer* **35**: 715–720

Lashford LS, Davies AG, Richardson RB, Bourne SP, Bullimore JA, Eckert H, Kemshead, JT and Coakham HB (1988) A pilot study of 131I monoclonal antibodies in the therapy of leptomeningeal tumours. *Cancer* **61**: 857–868

Marshall D, Pedley RB, Boden JA, Boden R and Begent RH (1994) Clearance of circulating radio-antibodies using streptavidin or second antibodies in a xenograft model. *Br J Cancer* **69**: 502–507

MIRD (1971) Medical Internal Radiation Dose Committee. *J Nuclear Med* **12**: 1–32

Moseley RP, Davies AG, Richardson RB, Kemshead JT, Coakham HB and Lashford, LS (1990) Intrathecal administration of 131 I radiolabelled antibody as a treatment for neoplastic meningitis. *Br J Cancer* **62**: 637–642

Papanastassiou V, Pizer BL, Chandler CL, Zananiri TF, Kemshead JT and Hopkins KI (1995) Pharmacokinetics and dose estimates following intrathecal administration of 131 I monoclonal antibodies for the treatment of central nervous system malignancies. *Int J Radiat Oncol Biol Phys* **31**: 541–552

Pizer B, Papanastassiou V, Hancock J, Cassano W, Coakham HB and Kemshead JT (1991) A pilot study of monoclonal targeted radiotherapy in the treatment of central nervous system leukaemia in children. *Br J Haematol* **77**: 466–472

Voute PA, Hoefnagel CA, de Kraker J, Valdes Olmos R, Bakker DJ and van de Kleij AJ (1991) Results of treatment with 131 I-metaiodobenzylguanidine (131 I-MIBG) in patients with neuroblastoma. Future prospects of zetotherapy. *Prog Clin Biol Res* **366**: 439–445

*© Cancer Research Campaign 1998*